# An Electrochemical Electrode to Detect Theophylline Based on Copper Oxide Nanoparticles Composited with Graphene Oxide

**DOI:** 10.3390/mi13081166

**Published:** 2022-07-23

**Authors:** Vinoda B. Patil, Shweta J. Malode, Sumitra N. Mangasuli, Suresh M. Tuwar, Kunal Mondal, Nagaraj P. Shetti

**Affiliations:** 1 Department of Chemistry, Karnatak Science College, Dharwad 580001, Karnataka, India; vinodapatil95@gmail.com (V.B.P.); sumamangasuli1818@gmail.com (S.N.M.); 2 Department of Chemistry, School of Advanced Sciences, KLE Technological University, Vidyanagar, Hubballi 580031, Karnataka, India; shweta.malode@kletech.ac.in; 3 Idaho National Laboratory, Idaho Falls, ID 83415, USA

**Keywords:** theophylline, graphene oxide, CuO nanoparticles, voltammetry techniques, limit of detection

## Abstract

The electrochemical analysis of theophylline (THP) was investigated by fabricating a carbon paste electrode (CPE) modified with graphene oxide (GO) along with copper oxide (CuO) nanoparticles (CuO-GO/CPE). The impact of electro-kinetic parameters such as the heterogeneous rate constant, the scan rate, the accumulation time, the pH, the transfer coefficient, and the number of electrons and protons transferred into the electro-oxidation mechanism of THP has been studied utilizing electrochemical methods such as cyclic voltammetry (CV) and differential pulse voltammetry (DPV). The differential pulse voltammetry technique was employed to investigate THP in pharmaceutical and biological samples, confirming the limit of detection (LOD) and quantification (LOQ) of the THP. X-ray diffraction (XRD) and scanning electron microscopy (SEM) analysis were performed to characterize the CuO nanoparticles. The CuO-GO/CPE was more sensitive in THP detection because its electrocatalytic characteristics displayed an enhanced peak current in the 0.2 M supporting electrolyte of pH 6.0, proving the excellent sensing functioning of the modified electrode.

## 1. Introduction

Theophylline (THP) is a product of xanthine and is chemically known as 1,3-dimethylxanthine. THP is mainly recommended for breathing problems and various asthmatic problems. THP has been commonly used as a breathing stimulant for chronic difficulty with breathing, moderate asthma in children, bronchiectasis, chronic obstructive pulmonary disorders, and adult emphysema [1,2]. When the level of THP rises above the concentration range of 20 μg/mL in plasma, it causes side effects including nausea, tachycardia, diarrhea, and disturbance in the central nervous system. It is an antagonist of adenosine receptors and phosphodiesterase inhibitors [3]. To avoid undesirable effects, it is necessary to set up an effective method for tracking THP from the lowest levels of plasma concentrations.

The electrochemical techniques used in pharmaceutical, textile, and food-related industries; ecological examinations; agriculture; and health care services have been successful in recent decades [4,5,6,7,8]. These methods retain advantages such as having greater sensitivity and selectivity, being user-friendly, being easy to use, being relatively cheap, being faster, and being applicable for different uses [9,10,11]. There are also several complications associated with using bare electrodes, such as slow electron transfer, the fouling of electrodes, etc. Given solving these problems, modifications are necessary. Modifiers, mainly nanomaterials, nanocrystals, and dyes, can decrease the excess potential required for the transmission of electrons and enhance the sensitivity and selectivity, thereby making them excellent contributors to electrochemical reactions [12,13,14].

The surface of the working electrode can be effectively modified by utilizing different modifiers such as surfactants, inorganic complexes, polymer conductors, metal-based nanoparticles, and clay particles [15,16,17]. This modified electrode involves an easy and less expensive preparation method, provides less background current in a wide range of potential windows and is very easy to adapt [18]. Hence, in the present study, we used a carbon paste electrode modified with modifiers such as CuO and GO to investigate the THP molecules.

Graphene is a two-dimensional honeycomb structure made of one-atom-thick hexagonal arrays of sp^2^-bonded carbon atoms. Due to its unique nanostructure and remarkable qualities, such as excellent conductivity, a vast surface area, and lower manufacturing costs, graphene has received much attention in many science and technology sectors [19,20]. Graphene has applications in the field of corrosion [21], nanodevices [22], biology [23], and energy materials [24]. It has also been extensively employed in fabricating electrochemical electrodes to identify organic molecules, biomolecules, and pharmaceutical compounds. Graphene is a vital component in electrochemistry-related applications because it has the characteristics of a single planar structure [25,26]. Graphene oxide (GO) is a graphene derivative containing varying oxygen, carbon, and hydrogen ratios. The oxidation degree of graphene oxide is affected by functional groups such as epoxy, hydroxy, and carboxyl on the GO’s surface. In water, GO has a high level of hydrophilicity and dispersibility. Electroactive biomolecules can be attracted or repelled by the negatively-charged GO through electrostatic interactions.

The CuO nanoparticles comprise a monoclinic structure and are a p-type semiconductor with a narrow band gap. These can exhibit a wide potential window, excellent catalytic properties, effective surface areas, super thermal conductivity, antimicrobial activity, and photovoltaic properties. Due to these properties, CuO nanoparticles are used in gas electrodes [27], as catalysts [28], as photo-electric chemical materials [29], in magnetic recording media, as supercapacitors [30], as high-efficiency thermal conducting material [31], as field emission emitters [32], etc. The CuO nanoparticles have been utilized mainly in working electrodes as electrocatalysts because of their vast surface area. The graphene oxide tolerance resulting from the collection and restacking through a van der Waals interaction decreased the electrode surface area. However, combining graphene oxide with CuO nanoparticles exhibited a higher capacitance than the individual materials. The composite of GO and CuO showed an outstanding rate capability compared with the other electrodes [33].

Different techniques have been applied to determine the presence of THP [34,35,36,37,38,39,40,41,42,43]. These approaches involve using other electrodes to examine THP. In the present study, the GO and CuO nanoparticles were utilized as modifier materials to fabricate an electrode that would determine the presence of THP and check its applicability in pharmaceutical and biological samples. As per the literature, no work has been reported on the investigation of THP using CuO-GO/CPE. The developed electrode under examination showed excellent reproducibility, sensitivity, and selectivity with a low detection limit in the analysis of THP.

## 2. Experimental Setup

### 2.1. Reagents and Chemicals

GO, CuO, and THP were procured from Sigma-Aldrich. The THP (0.1 mM) standard stock solution was prepared utilizing 99% pure ethanol. The analytical-grade KH_2_PO_4_, H_3_PO_4_, and Na_2_HPO_4_ were used to prepare the 0.2 M ionic strength of a phosphate buffer solution (PBS) in the pH between 3.0 and 9.2 as per the earlier reports. For all experimental investigations, double-distilled water was used.

### 2.2. Instrumentation

The electrochemical examination was executed using the electrochemical workstation model CHI-1112C (USA), which consisted of a three-electrode system; an Ag/AgCl (3.0 M) electrode as the reference electrode; a platinum wire as a counter electrode; and CuO-GO as modified working electrodes immersed in a 10 mL electrochemical cell. The pH meter (EQ-611, Equiptronics, Mumbai, India) was used to test the prepared pH value of the PBS, and for scanning electron microscopy analysis, a Model JEOL (SEM Model- jeol JSM-IT500LA, Tokyo, Japan) was utilized.

### 2.3. Preparation of the Working Sensor

The bare CPE was prepared with the help of graphite powder and paraffin oil with a ratio of 7:3. These were blended using a mortar and pestle. The prepared paste was kept for roughly 24 h to attain homogeneity. The paste was packed into a polytetrafluoroethylene tube (PTFE), and its surface layer was smoothed with some filter paper to obtain a shining and smooth electrode surface. Similarly, the modified electrode paste was prepared using graphite powder, paraffin oil, graphene oxide (0.05 mg), and copper oxide (0.05 mg). The modified paste was packed into a PTFE tube to obtain a CuO-GO modified CPE. Using cyclic voltammetry at a 0.05 s^−1^ scan rate, the voltammograms were recorded with a potential between 0.8 V and 1.5 V in the 0.2 M supporting electrolytes at a pH of 6.0. All experimental measurements were recorded at room temperature. After each measure, the carbon paste was carefully removed from the working electrode, and fresh, homogeneous paste was packed into the PTFE tube.

### 2.4. Preparation of the Excipients Solution

The different excipient test solutions were prepared to analyze the effect of the excipient interference on the electrochemical performance of THP. A standard stock solution was produced by dissolving a required quantity of the excipients in 100 mL of double-distilled water. This was followed by sonication for 10 min to obtain a perfectly dissolved solution. The prepared test solution was transferred to an electrochemical cell, and the investigations were carried out to analyze the excipients’ interference. Similarly, metal ion solutions were prepared.

### 2.5. Pharmaceutical Sample Preparation

The THP tablets were placed in a mortar and pulverized into a fine powder. A small amount of the fine powder weighing 0.1 mM was dissolved in the THP stock solution using ethanol. For the complete dissolution of the tablet, sonication was carried out for 10 min, followed by dilution in 100 mL of double-distilled water. An appropriate concentration of the tablet solution was examined, employing the DPV technique. The quantity of THP in the tablet was determined utilizing a calibration plot.

### 2.6. Urine Sample Preparation

In this study, urine samples were collected from volunteers. At the optimal temperature (25 ± 0.1 °C), the samples were centrifuged for 5 min. Further, the samples were diluted using an electrolyte solution. Then, a known concentration of the THP solution was added to the urine samples to prepare spiked urine samples. Based on the DPV approach, the recovery of THP in urine samples was estimated.

## 3. Results and Discussion

### 3.1. The Surface Area of the Electrode

**Figure 1:**
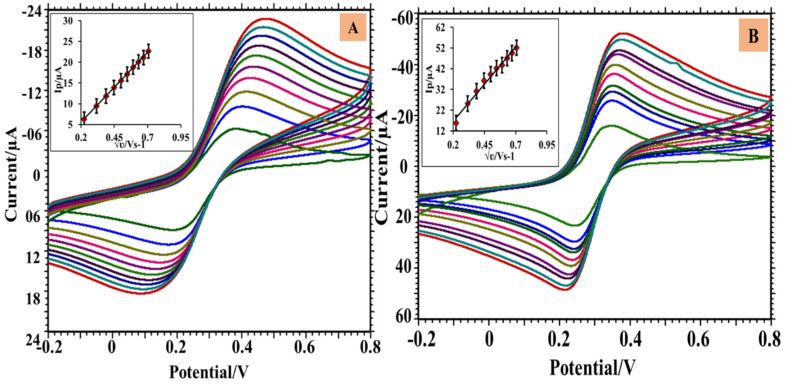
Cyclic voltammogram of K_3_[Fe(CN)_6_] (1.0 mM) at different scan rates from 0.05 to 0.5 Vs^−1^. (**A**) At bare CPE. (**B**) At CuO-GO/CPE.

In the electrochemical analysis, the surface of the electrode plays a significant role as the electrochemical signals derive from the analyte molecule and the electrode interaction depends on the active area of the electrode. The surface area can affect the sensitivity of the electrode material. Hence, the surface area of the electrode was measured by utilizing a CV approach for the K_3_[Fe(CN)_6_] (1.0 mM) test solution in a KCl (0.1 M) electrolyte. The cyclic voltammograms were obtained by varying the scan rate values. The Randles–Sevcik Equation (1) was employed to evaluate the active surface area using the slope value from the plot of Ip vs. √ν [44]. A cyclic voltammogram obtained by varying the scan rate and the slope value from the plot of I_p_ vs. √ν and by employing the Randles–Sevcik Equation (1) was used to evaluate the active surface area [44]. The linear regression equations are I_p_ = 33.56√ν − 1.157; R^2^ = 0.999 and I_p_ = 71.61√ν − 2.319; R^2^ = 0.986 for the bare CPE and CuO-GO/CPE, respectively (Figure 1). The surface areas of the electrode were found to be 0.045 cm^2^ for the bare CPE and 0.096 cm^2^ for the CuO-GO/CPE. The obtained value shows a nearly two-fold enhancement of the surface, providing the effective modification of the electrode by including the CuO and the GO. The geometrical surface area of the utilized electrode was 0.09 cm^2^, comparable with the obtained active surface area of the modified electrode.

(1) $$I_{p}=\left(2.69\times 10^{5}\right)n^{3/2}{\mathrm{ADo}\;}^{3/2}C^{1/2}$$

### 3.2. Characterization of the Modifier

**Figure 2:**
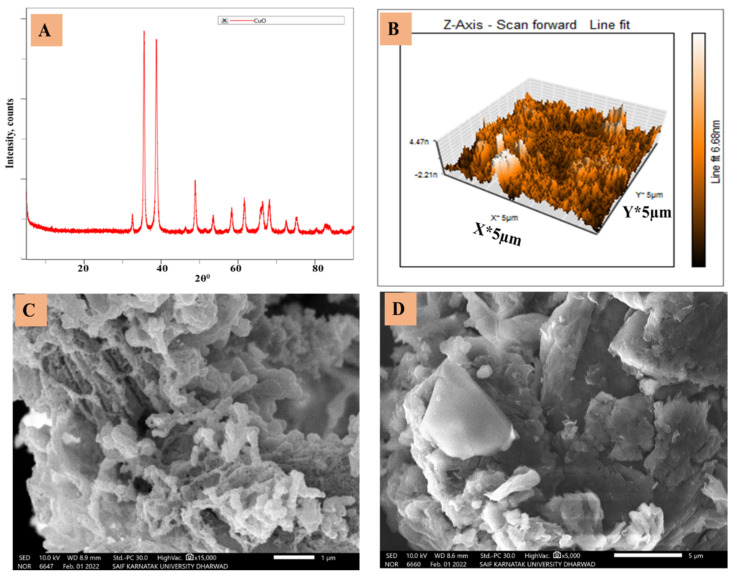
(**A**) XRD of the CuO, (**B**) AFM of the CuO-GO/CPE, (**C**) SEM image of the CuO, and (**D**) SEM image of the GO.

The XRD pattern of the CuO nanoparticles, displayed in Figure 2A, shows a monoclinic single-phase structure. The diffraction peaks of the CuO nanoparticles are at 2θ = 35.53° and 38.67°. The peak intensity and positions of the peaks are very close to the earlier reported values. The diffraction peaks are broad due to the nanostructure of the CuO nanoparticles. The AFM analysis of the modified electrode revealed in Figure 2B, exhibited an improved surface roughness of the modified electrode. As shown in the SEM image (Figure 2C), the CuO nanoparticles formed an irregular planar structure. The test sample possessed a platter-like shape, which was neither amorphous nor a crystal structure, as depicted in the SEM image of the GO in Figure 2D. By its flaky texture, the surface morphology of the GO was characterized. The SEM image displays the edges of the GO that are thinner and more prominent in the interspace.

### 3.3. Electrochemical Behavior of THP

**Figure 3:**
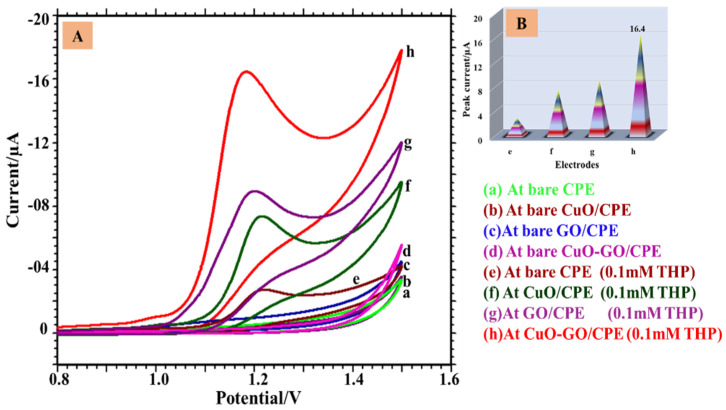
(**A**) Cyclic voltammograms of 0.1 mM THP in pH 6.0 of the PBS at 0.05 Vs^−1^. (**B**) Deflection of peak current in the CPE, CuO/CPE, GO/CPE, and CuO-GO/CPE.

The cyclic voltammetry approach was used to monitor the electrochemical behavior of the THP in the working electrodes using a PB solution with a pH of 6.0. The cyclic voltammograms were recorded at a 0.05 Vs^−1^ scan rate for the 0.1 mM THP and the bare and modified CPE. From a comparative study (Figure 3A), we observed a considerable conversion in the cyclic voltammogram of the 0.1 mM THP. The electro-oxidation peak with a current value of 2.73 µA was noticed in the CPE, 8.91 µA in the GO/CPE, 7.29 µA in the CuO/CPE, and 16.40 µA in the CuO-GO/CPE. Nanoparticles of the CuO were doped with the GO using its functional groups as adsorptive sites. Further, the hydrophilic properties of the GO are caused by their polar oxygen functional groups. According to reports, the oxygen elements of the GO can stabilize the adsorbed CuO nanoparticles, which act as a nucleation center for the metal nanoparticles. Including CuO nanoparticles and the GO in preparing the electrodes showed the maximum peak intensity compared to the bare CPE, GO/CPE, and the CuO/CPE due to the large surface of the CuO and the GO. A well-oriented enhanced peak was detected in the CuO-GO/CPE (Figure 3B), which proves the successful modification of the CPE. The area of the modified CPE was important for enhancing selectivity and sensitivity. Thus, the modified CuO-GO/CPE was utilized for further investigation. Further, a reduction peak was not observed in the reverse scan, suggesting that the process was irreversible in the electrode.

### 3.4. Effect of the Accumulation Time

**Figure 4:**
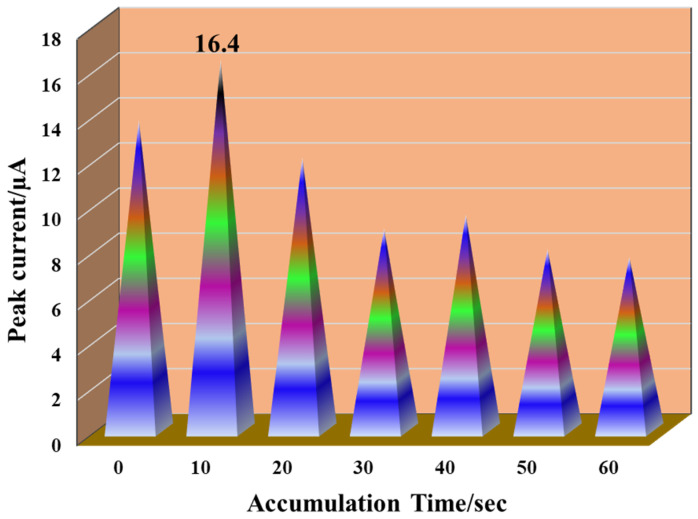
Effect of the accumulation time on THP (0.1 mM) in the CuO-GO/CPE.

The electrochemical oxidation of the 0.1 mM THP on the electrode’s surface depended on the accumulation time between the THP and the surface of the electrode, which indicated that the concentration of the THP molecule may have affected the electrochemical activity in the vicinity of the electrode. The cyclic voltammetry technique was employed to study the accumulation time, and we observed that the voltammograms for the 0.1 mM THP concentration were from 0 s to 60 s. The detailed studies of the impact of the accumulation time on the oxidation peak current are shown in Figure 4. We observed a well-oriented and intensified peak at 10 s, due to the high concentration of analyte molecules aggregating near the surface of the electrode, causing the peak current to increase, directly affecting the electrode’s sensitivity. Further, after 10 s of accumulation time, the saturation limit was reached, and the peak current decreased. However, a maximum peak intensity was observed for 10 s of the accumulation time; hence, the same was used as the optimum for further parameter examinations.

### 3.5. Variation in the PH

**Figure 5:**
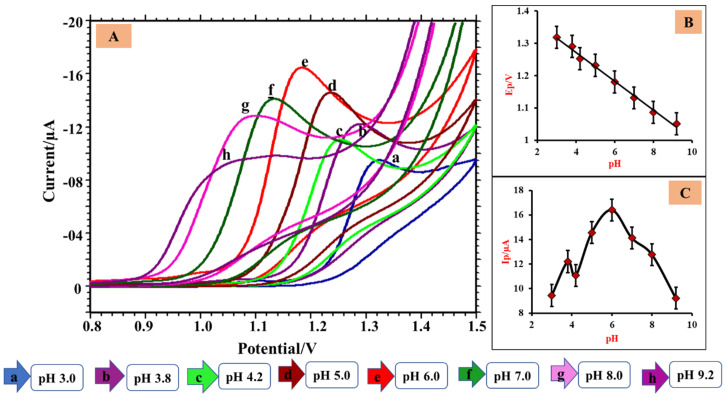
(**A**) Cyclic voltammograms of the THP (0.1 mM) in different electrolytic solutions in the CuO-GO/CPE; t_acc_ = 10 s; scan rate = 0.05 Vs^−1^. (**B**) Plot of E_pa_ vs. pH. (**C**) Change in peak current with the pH.

In the electrochemical examination, the supporting electrolytic solutions of various pH values play a vital role. The peak current and potential values depend on the supporting electrolytes of different pHs. Thus, it was essential to investigate the impact of the pH of the supporting electrolytes on THP to conclude the optimal pH. In this electrochemical investigation, the CV technique was utilized, and cyclic voltammograms were recorded with a potential window between 0.8 V and1.5 V. Figure 5A displays the impact of a PB solution of 0.2 M for pH ranges varying from 3.0 to 9.2 in a 0.1 mM THP solution. Figure 5B shows that with the increasing pH value, the potential values of the THP shifted to less potential, pointing to the participation of protons in the electrochemical oxidation process [45]. From the plot pH vs. E_pa_, we attained the regression equation: E_pa_ (V) = −0.044 pH + 1.449; R^2^ = 0.993. In this investigation, the slope value −0.044 V/pH is closer to the standard Nernstian value of 0.059 V/pH, indicating the same number of proton and electron participation in the electro-oxidation process [46]. At pH 6.0, we obtained the highest peak current (Figure 5C). Hence, pH 6.0 was optimized for further investigation.

### 3.6. Influence of the Scan Rate

**Figure 6:**
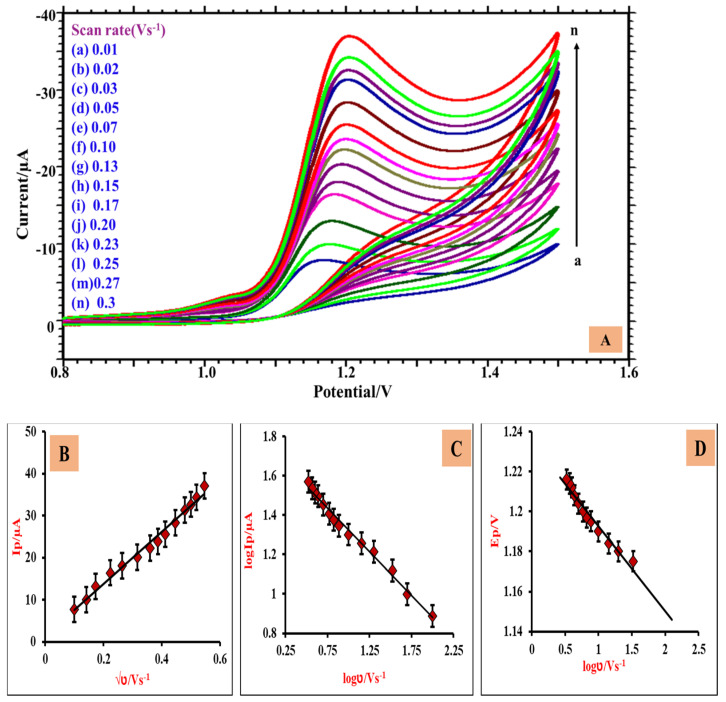
(**A**) Cyclic voltammograms of the THP (0.1 mM) in the PBS of pH 6.0 for the varying scan rates from 0.01 Vs^−1^ to 0.3 Vs^−1^; t_acc_ =10 s. (**B**) Plot of I_p_ (µA) on √ט. (**C**) Plot of log I_p_ vs. log υ. (**D**) Plot of E_p_ vs. log υ.

Physicochemical parameters such as electron participation, electron transfer coefficient, and rate constant can be calculated with the help of scan rate studies. The electrochemical examination was performed utilizing the CV technique by changing the scan rate from 0.01 Vs^−1^ to 0.3 Vs^−1^, as shown in Figure 6A. The linear relationship between the peak current (I_pa_) and the square root of the scan rate (√ט) has been noticed in Figure 6B, with a linear regression equation: I_p_ (μA) = 61.906 √ט +1.376; R^2^ = 0.986, which indicates that the THP experienced a surface diffusion electrochemical oxidation process in the CuO-GO/CPE [47]. From the graphic presentation of log I_pa_ vs. log ט (Figure 6C), we have attained the linear regression equation; log I_p_ = −0.444 log 1.769 + ט; R^2^ = 0.989. The slope value of 0.44 nearly equals the expected value of 0.5, indicating the diffusion-controlled electrochemical process [48]. The plot of E_pa_ vs. log ט is shown in Figure 6D with a linear regression equation: E_pa_ (V) = −0.056 log 1.230 + ט; R^2^ = 0.961.

The Laviron Equation (3) [49] was utilized in the irreversible electrochemical oxidation process to calculate the heterogeneous rate constant value. As the scan rate increases, the potential of the peak shifts towards a positive value, confirming that the electro-oxidation process was irreversible. Using the Bard–Faulkner Equation (2) for the irreversible process, the charge transfer coefficient (α) was calculated to be 0.55.

(2) $$E_{p}-E_{p}/2=\left(\frac{47.7}{\alpha }\right)\mathrm{mV}$$

(3) $$E_{p}=E^{0}+\left(\frac{2.303RT}{\left(1-\alpha \right)\mathrm{nF}}\right)\log\left(\frac{\left(1-\alpha \right)\mathrm{nF}}{{\mathrm{RTk}}^{0}}\right)+\left(\frac{2.303\mathrm{RT}}{\left(1-\alpha \right)\mathrm{nF}}\right)\log\;\upsilon$$

where E is the formal redox potential, k^0^ is the heterogeneous rate constant, n is the number of electrons involved in the electrochemical reaction, and other symbols represent standard descriptions. The value of n and k^0^ were calculated using the plot of E_pa_ vs. log ט. The heterogeneous rate constant was determined to be 4.49 s^−1^, and the number of electrons participating in the electrochemical oxidation of the THP in the modified electrode was computed to be 1.92 ≈ 2.0.

### 3.7. Plausible Reaction Mechanism of the THP

**Scheme 1:**
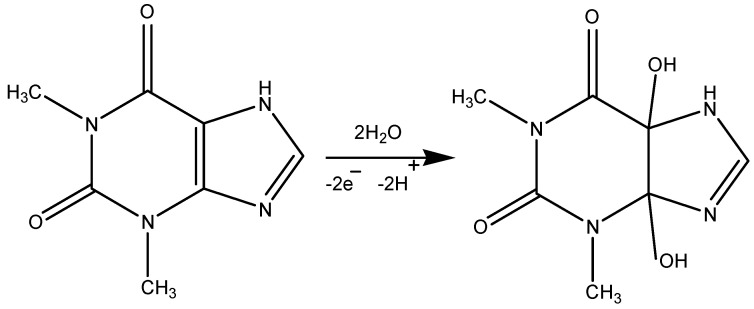
Possible mechanism of THP.

From the studies on the scan rate, it was noticed that the THP experiences an electrochemical oxidation reaction controlled by a diffusion process. This examination reveals that the number of participating electrons in the electrochemical reaction was two, according to the linear equation of E_pa_ vs. pH, involving an equal number of protons and electrons in the electrochemical mechanism [50]. The possible electro-oxidation mechanism of the THP is shown in Scheme 1.

## 4. Analytical Applications

### 4.1. Influence of Concentration Variation

**Figure 7:**
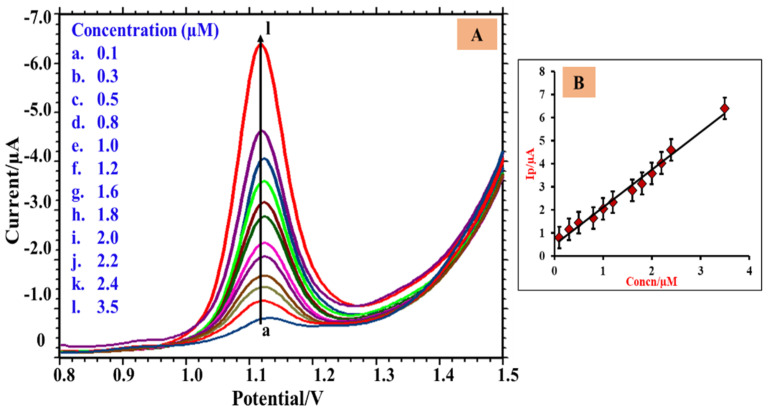
(**A**) Differential pulse voltammograms of the THP in the CuO-GO/CPE modified electrode; t_acc_ = 10 s. (**B**) Calibration plot of the peak current against the concentration.

The concentration of THP was analyzed using the DPV technique. The DPV investigations were performed by changing the THP concentration range from 0.1 to 3.5 µM. From Figure 7A, it can be visualized that the peak current increases with an increase in the concentration of the THP. The linear relationship between I_pa_ and the concentration from the calibration plot, as shown in Figure 7B, is I_pa_ = 1.627C (THP) + 0.475; R^2^ = 0.987. The detection limit and quantification were evaluated utilizing the following equations [51].

(4) LOD=3sm

(5) LOQ=10s/m

where m is the slope value from the calibration plot and s is the intercept of the standard deviation. The values for the limit of detection (LOD) and limit of quantification (LOQ) were evaluated to be 8.33 × 10^−9^ and 27.79 × 10^−9^, respectively. Table 1 gives a comparative investigation with the relative reported work.

### 4.2. Impact of the Excipients

**Figure 8:**
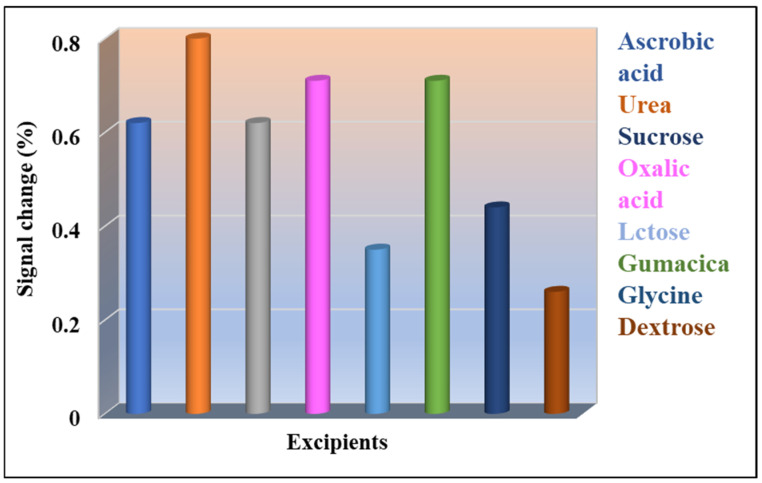
Effect of excipients on the potential values of the THP in the CuO-GO/CPE.

To investigate the interaction between THP and the excipients used in medical formulations, the most commonly used excipients were selected. A known concentration of THP and a hundred-fold excess concentration of excipients were prepared for this examination. The DPV technique was used to measure the shift in the potential values for the THP, and the absence and presence of excipients between the potential values of 0.8 and 1.5 V. Based on Figure 8, the excipients did not interfere with the THP oxidation signal as the change in peak potential was less than 5%.

### 4.3. Impact of Metal Ions

We also examined the influence of metal ions in the electroanalysis of THP. Table 2 shows the results of the complete investigation, and it was noticed that (NH_4_)_2_SO_4_, CuSO_4_, Cu(CH_3_COO)_2_, KNO_3_, etc., did not interfere with the peak signals of THP even at a hundred-fold excess concentration. The interference was within a tolerance limit of less than 5%, as shown in Table 2. As a result, the electrode was selective to the THP in the presence of metal ions.

### 4.4. Analysis of the Pharmaceutical Sample

The capability of the developed electrode was tested by a tablet sample examination. The tablet solution preparation method was provided in Section 2.5. The samples were examined by utilizing the DPV technique. The known quantity of the standard THP tablet samples was investigated and correlated with the results acquired from the concentration variation studies of known concentrations. The recovery value obtained was 98%, along with an RSD value of 2.21%. Table 3 summarizes the findings of the tablet analysis.

### 4.5. Analysis of the Urine Sample

The reliability of the CuO-GO/CPE electrodes was examined through a urine sample analysis. Urine sample solutions were prepared and mentioned in Section 2.6. A DPV method was utilized to calculate the recovery of THP in the human urine samples. The calibration plot was used to evaluate the unknown concentration of the analyte; the recovery values ranged from 95 to 98.75%. The recovery values show the efficiency of the developed electrode in determining the spiked THP in the urine sample. The obtained results are shown in Table 4.

### 4.6. Stability of the Developed Electrode

The repeatability measurements were performed to verify the stability of the developed electrode. The CuO-GO/CPE was stored in an airtight vessel for 6 days before analysis. The constructed electrode retained 91.91–99.26% of its initial response after 6 days of storage, providing excellent stability and capacity. The reproducibility was also one of the important factors in determining the efficacy of the developed sensor. The investigations were carried out to check the stability of the modified electrodes for more than one analysis. The CV measurements were taken for THP with the same paste as for the three respective trials, and it was noticed that the peak current declined gradually after the second and third measurements. Hence, washing the electrode and using fresh paste for each measurement was recommended to obtain reproducible results. The sensor reproducibility was examined by taking five consecutive measurements (using a new paste and after pretreatment for every measurement). The results showed 1.17% of the RSD, proving the extraordinary reproducibility nature of the proposed sensor material. Furthermore, the CuO-GO/CPE shows higher stability and reproducibility, suggesting that the sensor can be applied in analytical applications.

## 5. Conclusions

The cyclic voltammetry and differential pulse voltammetry techniques have successfully identified THP in the fabricated CuO-GO/CPE. Compared to the bare CPE, the CuO-GO/CPE has greater sensitivity and a faster electron transfer rate. From the influence of the pH study, it was noticed that pH 6.0 was optimal for further investigations. The THP experienced an irreversible electrochemical process involving the transfer of two protons and two electrons on the electrode surface and controlled via a diffusion process. The physicochemical parameter was evaluated by examining the scan rate variation. The CuO-GO/CPE electrode shows a lower detection limit (8.33 × 10^−9^ M), superior sensitivity, and greater selectivity. THP may also be detected in pharmaceutical and urine samples using the modified electrode. An interference examination indicated that the excipients and metal ions do not affect the oxidation signal of the THP, thus revealing the excellent selectivity of the electrode.

## Figures and Tables

**Table 1:** Comparison of detection limits.

Sl. No.	Electrodes	Linearity Range	Detection Limit	Reference
1	MWNTs/Au/poly-L-lysine/SPE	10–200 μM	2.0 × 10^−6^ M	[34]
2	CdSe/GCE	1.0–700 µM	4.0 × 10^−7^ M	[35]
3	poly(H-A)/GCE	0.4–17 μM	3.2 × 10^−7^ M	[36]
4	P(L-Asp)/f-MWCNTs/GCE	0.1–50 μM	2.0 × 10^−7^ M	[37]
5	GQDs/SPE	1.0–700.0 μM	2.0 × 10^−7^ M	[38]
6	Poly(CTAB)/GCE	0.5–1000 μM	1.1 × 10^−7^ M	[39]
7	MnO_2_ nanosheets/IL-graphene/GCE	1–220 μM	1.0 × 10^−7^ M	[40]
8	AuNPs/aptaelectrode	0.1–80 μM	7.0 × 10^−8^ M	[41]
9	MWCNTs/GCE	0.3–10 μM	5.0 × 10^−8^ M	[42]
10	PLCY/N-CNT/GCE	0.10–70.0 μM	3.8 × 10^−8^ M	[43]
11	CuO-GO/CPE	0.1–3.5 µM	8.33 × 10^−9^ M	Present method

**Table 2:** Metal ion study on THP.

Metal Ions	Potential Observed (V)	Potential Difference (V)	Signal Change (%)
(NH_4_)_2_SO_4_	1.128	0.006	0.53
CuSO_4_	1.130	0.008	0.71
Cu(CH_3_COO)_2_	1.125	0.003	0.26
KNO_3_	1.119	−0.003	−0.26
NH_4_Cl	1.127	0.005	0.44
BaCl_2_	1.129	0.007	0.62
KCl	1.120	−0.002	−0.17
NaCl	1.128	0.006	0.53

**Table 3:** Analysis of the tablet sample.

THP	Observations
Labeled claim (mg)	300
Obtained amount(mg) ^a^	294
RSD%	2.21
Added (mg) ^a^	1.0
Obtained (mg)	0.98
Recovery%	98

^a^ (Average of three determinations).

**Table 4:** Urine sample analysis.

Urine Samples	Added (10^−6^ M)	Obtained (10^−6^ M)	Recovery (%)
1	2.0	1.90	95.00
2	1.8	1.77	98.75
3	1.6	1.56	97.50

(Average of three determinations).

## Data Availability

Not applicable.
